# Decolonising Canadian water governance: lessons from Indigenous case studies

**DOI:** 10.14324/111.444/ucloe.000060

**Published:** 2023-06-23

**Authors:** Corey McKibbin

**Affiliations:** 1 Department of Philosophy, McMaster University, Hamilton, Canada

**Keywords:** decolonisation, water, policy, Indigenous, ethics

## Abstract

Meaningful lessons about decolonising water infrastructure (social, economic and political) can be learned if we scrutinise existing governance principles such as the ones provided by the Organisation for Economic Cooperation and Development in 2021’s Principles on Water Governance. Instead of using *only* Western frameworks to think about policy within Indigenous spheres of water, sanitation and hygiene, the Government of Canada can look to Indigenous ways of knowing to complement their understanding of how to govern areas of water, sanitation and hygiene efficiently. In this paper, the term *Indigenous* encompasses First Nations, Inuit and Métis populations. This paper is presented as *a* step out of many towards decolonising water governance in Canada, and is intended to show that it is necessary to make space for other voices in water governance. By highlighting the dangers in the case studies, three lessons are apparent: (1) there needs to be an addition of Indigenous Two-Eyed Seeing in water governance; (2) Canada must strengthen its nation-to-nation praxis with Indigenous communities; and (3) there needs to be a creation of space in water, sanitation and hygiene that fosters Indigenous voices. This is necessary such that there can be equal participation in policy conversations to mitigate existing problems and explore new possibilities.

## Introduction

Meaningful lessons about decolonising water infrastructure (social, economic and political) can be learned if we scrutinise existing governance principles such as the ones provided by the Organisation for Economic Cooperation and Development (OECD) Principles on Water Governance [1]. Instead of using *only* Western frameworks to think about policy within Indigenous spheres of water, sanitation and hygiene (WaSH), the Government of Canada can look to Indigenous ways of knowing to complement their understanding of how to govern areas of WaSH efficiently. For this paper, the term *Indigenous* encompasses First Nations, Inuit and Métis populations [2–4] (Metallic, 2022).^1^

While Indigenous communities in Canada make up approximately 4.3% of the overall population, they, unfortunately, face a large proportion of the water crises in Canada [2]. According to the Auditor General of Canada, one in five Indigenous communities are under ‘water advisories’ (meaning the water is not safe for use), and ‘more than half of water systems in the lands reserved for Indigenous people posed a medium or high risk’ [2,5]. These comments have resulted in Canada’s Indigenous land reserves being compared to low-to-middle-income countries [2].

I argue that the problems within water governance, in Indigenous spheres, are due to colonisation. Simms et al. [6] points out that existing issues in WaSH are systemic and due to supposed provincial and federal government ownership over all water in Canada [6].^2^ The federal and provincial governments laying claim to water trivialises the ‘ethic of responsibility’ that many Indigenous populations adhere to when interacting with water [8]. McGregor [8] argues that ‘the major strength of First Nations involvement and input [in water governance] is the consideration of values, ethics, and knowledge that provide a holistic understanding of water’ (clarification added; [8]). A similar sentiment is issued by Simms et al. [6] who write that many Indigenous communities view water as something spiritual, medicinal and the ‘life-blood of the land’ [6]. Rather than reframe existing water governance principles using Western methodologies in areas of WaSH, there must be equal participation of Indigenous expertise and ways of knowing such that altered principles can be adhered to by all transparently.

This paper aims to critique two of the 12 OECD’s Principles on Water Governance that the Government of Canada supports to promote decolonisation within Canadian water governance. In the What does it mean to decolonise water? section, I give a brief description of water policy in the Canadian context to help ground readers. Specifically, I introduce the Canada Water Act. Then, I raise an important question: what does it mean to decolonise Canadian water policy? In the Thinking otherwise: re-examining the OECD Principles on water governance section, I make headway on this question: I give a brief history and overview of the OECD Principles on Water Governance which Canada supports. I then present the Indigenous research methodology of Two-Eyed Seeing. By presenting this research methodology, I aim to show that it is possible to decolonise water in a meaningful way, but in order to do so, greater attention and appreciation for Indigenous knowledge systems needs to be present within policy. In the Towards decolonisation section, I introduce three case studies from three separate categories of Indigenous organisation across Canada. These case studies showcase the discrepancy of power between Canada and Indigenous Nations in contemporary water policy. In the Call-to-action section I end on an emancipatory note by critiquing two of the OECD Principles. I critique Principles Four and Nine as, I argue, they reflect the current inability of the Canadian government to effectively deal with Indigenous water issues. I conclude this paper by arguing that if Canada takes these critiques into consideration, then there can be a decolonisation of water policy; however, to decolonise water effectively, Canada needs to take seriously the lessons learned in this paper when forming new policies. If Canada does not take these lessons into consideration, what might come otherwise will be more of the same.

## What does it mean to decolonise water?

### The role of the Canada Water Act in Canadian water policy

In this section, I will explain what I mean by decolonising water in the Canadian context. To do this, I will expand on the role of the Canada Water Act and how it impacts policy at the federal and provincial levels. Understanding how the Canada Water Act impacts policy at the federal and provincial levels will reveal that ownership claims of water on behalf of the Canadian government impact Indigenous persons’ ability to self-determine. This, in turn, shapes the colonisation of water (expanded on below in the next section).

Originally instated in 1970, the Canada Water Act was the Canadian government’s way to ensure the cooperation of provinces and territories to deliver and develop adequate water infrastructure, sanitation and hygiene across the country [9]. Every year since the instantiation of the Act, it has been mandated that a report be made on behalf of provinces to be presented to the Parliament of Canada by the end of the fiscal year [10]. These reports are to outline and provide updates about water within provinces and territories for the Federal government [10]. These reports also include information pertinent to ongoing water research and tracks ‘the development and use of Canada’s water resources’ [10]. Therefore, the Act is essential to understand how the Canadian government interacts with water at both the national scale (and, to an extent, the international scale as will be discussed below). The Act also legitimises the Canadian government’s claim to ownership of water and its ability to track how the provinces are developing infrastructure and governance strategies related to water. Thus, the Act is necessary for understanding how water policy impacts *all* Canadians. In this way, it is necessary to note the interrelatedness of political ideas such as infrastructure, governance and policy, which might at first seem disparate. As we can see from this brief description of the Canada Water Act, they are highly interrelated, and each is important for the maintenance of the other. The Act, which is a set of policies, impacts the provincial governance of water and the kinds of infrastructure developed within the governance strategies.

Pointing out the significance of the Canada Water Act and its accompanying yearly reports also highlights another convoluted aspect of water governance in Canada. It might seem that the provinces control much of the narrative concerning water governance: after all, provinces often control the resources within their borders. However, the amount of autonomy that provinces and territories have over resources is likely to differ depending on their borders and other geopolitical scenarios. For example, the ocean water of the Northwest Passage (NWP) in Nunavut has variable levels of governance influence from the Inuit Circumpolar Council, the federal government, the governments of the territories and international governments and agencies outside of Canada that claim ownership and regulatory power of the NWP ([11] provides a comprehensive analysis of the national and international relations and claims over the NWP). Similarly, bodies of freshwater in the Niagara region of Ontario span transnational, national and provincial jurisdictions. In writing about these different kinds of waters and scales, it is essential to keep in mind the Canada Water Act and its associated documentation when writing about Canadian water policy. Similarly, it is also important to note how the federal government claiming ownership of water has impacted Indigenous persons’ rights to self-determination with regards to resources in their Treaty territory (see end note for more information about Treaty rights). Therefore, we must think about how to decolonise water in a way that respects Indigenous persons’ agency while securing the rights of water for all.

### Decolonising water

Thus, an important question remains: what does it mean to decolonise water policy in the Canadian context? In this article, I am using decolonisation in a similar way that Taylor et al. [12] are: I am referring to the right and recognition of Indigenous people to govern their water and lands without or with minimal federal or provincial interference. Some Indigenous Nations have made progress in this regard. For example, the Syilx Nation Water Declaration argues:

All life requires siwɬk^w^ and yet our siwɬk^w^ supplies are quickly becoming over allocated, abused and polluted. Challenges related to siwɬk^w^ quality, access, quantity, use and allocation have become more prevalent within Syilx Territory. siwɬk^w^ is not being respected under externally driven government regulations and management conditions. Syilx People question not only the provincial and federal government’s decision-making authority related to the use of our siwɬk^w^ but also their practices. Syilx Nation [13]

It is useful to note that *siwɬk^w^* is the *nsyilxcən* word for water. Importantly, this declaration refers to the federal government and provincial government of British Columbia’s failed involvement in water governance. As such, it speaks directly to the goals of the rest of this paper: how can we fruitfully think otherwise (i.e., differently) about how to approach water in the national and international contexts by scrutinising international instruments such as the OECD to conceive of equitable access to water for all Canadians?

## Thinking otherwise: re-examining the OECD Principles of water governance

### History of the OECD and water colonialism

The history of the OECD starts at the end of World War II when the Organisation for European Economic Cooperation (OEEC) was conceived in 1948 [12]. In 1960, the OEEC extended into North America, when Canada and the United States joined, and to Australasia when New Zealand and Australia joined [12]. This is important to note as those four countries are often linked with British imperialism and hence the mitigation and termination of Indigenous and Aboriginal epistemes in North America and Australasia, respectively [12]. The creation of the OEEC and subsequent development of the OECD after membership extended outside of Europe are linked to colonialism. The kind of colonialism important in the context of this paper is termed *water colonialism* [12]. Water colonialism includes the ‘dispossession, denial or erasure, of Indigenous peoples’ management and water diversion, pollution of water as a result of states activities, destruction of water places, and inadequate drinking water and sanitation service delivery’ [12].

Water colonialism is in conflict with the mission of other UN organisations such as the United Nations Declaration on the Rights of Indigenous Peoples (UNDRIP). UNDRIP urges states to recognise the rights of Indigenous peoples in member countries. In the context of water, UNDRIP recognises that Indigenous groups’ claim to water is ‘substantial’ and that ‘Indigenous peoples have the right to their own water laws, systems of water governance, and institutions, consistent with their own frameworks for water management’ [12]. As such, decolonisation of water governance in OECD member countries is paramount to the recognition of Indigenous rights.

### OECD Principles

Instated in 2009 by the OECD, a multi-national governmental organisation dedicated to economic policy, the OECD Principles on Water Governance were created to ‘identify and help governments, at all levels, bridge critical governance gaps in the design and implementation of their water policies’ [14]. The OECD Principles focus on the ‘economic analysis’ and ‘best practices’ of governing water in various countries, utilising language that is antithetical to Indigenous ways of perceiving and acting with water [14]. The OECD’s consideration of ‘territorial development’ positions water management, *not* as an environmental issue, but as one of economics [14].^3^

The OECD Principles are promoted as an analytic tool that countries can use to formulate ‘neutral and flexible’ solutions to water policy [12]. Emphasising the neutrality of the OECD Principles is important as they are promoted as place-based and context-dependent [12]. Therefore, while a country can use these principles as a guiding framework for how to deal with water governance, they must be scrutinised if they are to be used effectively in context-specific settings. It is important to note that the OECD Principles on Water Governance stand on three pillars:

1. Effectiveness: A straightforward pillar that asks whether the Principles on Water Governance are effective or not in a government’s strategic layout of water policy [14].
2. Efficiency: This pillar states that an efficient water plan comes with the least cost for society [14]. However, when there are two opposing ideals at play – water as an economic resource and water as lifeblood – it is difficult to consider who is supposed to endure the least costs. If both societies (the colonised and the dominant) are to share equal costs, it is unclear from the principles how that is supposed to happen.
3. Trust and engagement: The governments that employ the OECD Principles (including Canada), must build confidence and be inclusive with stakeholders [14]. Governments must act with integrity while monitoring, evaluating and adjusting water governance ‘when needed’ [14].

One way to achieve the goals of the OECD pillars is to include alternative ways of knowing in the conversations that shape policy. One alternative way of knowing within Indigenous research is the methodology of Two-Eyed Seeing. Two-Eyed Seeing can help evaluate at least two of the 12 OECD Principles. Specifically, I will look at Principles Four and Nine:

Principle Four: ‘Adapt the level of capacity of responsible authorities to the complexity of water challenges to be met, and to the set of competencies required to carry out their duties’ [1].

Principle Nine: ‘Mainstream integrity and transparency practices across water policies, water institutions and water governance frameworks for greater accountability and trust in decision-making’ [1].

Principles Four and Nine are the most urgent to be critiqued. As will be shown below in the case studies that are examined, there are serious gaps in trust between Indigenous and non-Indigenous communities. There is also a lack of understanding regarding the complexities surrounding areas of WaSH in Indigenous communities that provincial governments have yet to address efficiently. Principles Four and Nine have added importance as they point to the problematic notion of incommensurability in policy studies that is put forward in Laurence Tribe’s paper ‘Ways not to think about plastic trees’ [16]. Ethical considerations such as trust, integrity and other kinds of value-laden competencies are not easily enumerated and so are often thrown to the wayside in policymaking as subjective interferences. Tribe characterises these kinds of incommensurables as ‘fuzzy’ or ‘fragile’ and possibly problematic for policymakers that rely on ‘objective’ (i.e., numeric) data to make policy decisions [16]. However, relying on *objective* data is problematic for Indigenous communities, as spirituality linked to water, land and other natural resources cannot be easily quantified in a way that is meaningful for policymakers. Therefore, it is prudent to question governments that utilise the OECD as an analytic tool to truly conceive of responsible authority and act with trust and integrity in water governance in a way that is beneficial to Indigenous communities. Tribe argues that ‘we must therefore develop a new group of professionals sensitive to the sorts of values and issues that analyses currently tend to slight – diversity, balance, aesthetic quality, reversibility, the claims of the future – and adept at modelling policy impacts in terms of such values’ [16]. This claim is hasty: we do not need a group of new professionals to reckon with these non-commensurable values. Rather, we should look to local community members and learn from their pieces of knowledge to inform more robust policymaking.^4^ One way to do this is by incorporating Indigenous research methods such as Two-Eyed Seeing.

### Two-Eyed Seeing

To mitigate the lack of understanding about Indigenous water issues in areas of governance and to foster trust between settler and Indigenous communities, it is prudent that governments utilise an Indigenous research methodology such as Two-Eyed Seeing. Two-Eyed Seeing (*Etuaptmunk* in Mi’kmaw) was conceptualised by Mi’kmaw Elder Dr. Albert Marshall [18].^5^ Two-Eyed Seeing is a ‘model of knowledge’ that promotes the utilisation of both Western and Indigenous ways of knowing [18,20].^6^ Corroborated by McGregor, Andrea Reid and colleagues state that central to Two-Eyed Seeing is an idea of *Netukulimk*, or an ethic of responsibility [8,18]. Water scholars, practitioners and policymakers *must* pay attention to the potential harms of current practices and policies, and how they can impact future generations. Within Two-Eyed Seeing two principles are adhered to:

1. ‘Learn from one eye with the strengths of Indigenous knowledge and ways of knowing’ [20].
2. ‘From the other eye with the strength of Western knowledges and ways of knowing’ [20].

As noted by Reid et al. [18], it is not a question of ‘*whether*’ Two-Eyed Seeing can be utilised in areas of WaSH: it is a question of how governments can transform and shape their policies to recognise Indigenous methodologies as legitimate [18,23]. In other words, how can we apply Indigenous Two-Eyed Seeing to Canadian water governance practices? The next section will address this question by examining three case studies.

## Toward decolonisation

Here I will briefly refer to three Case Studies, taken from existing literature, that explain opportunities to fill water policy gaps within various levels of Indigenous organisation across Canada. Once the Case Studies have been explained, it will be made clear how water governance in Canada can move toward decolonisation.

### Key developments in Canadian policy and legislation concerning First Nations drinking water quality from 2003 to 2013

In 2003, the Government of Canada announced that it would create a First Nations Water Strategy [8]. The strategy would aim to utilise resources that could improve ‘the safety of water supplies in First Nations communities’ [8]. By 2008 it was evident that the strategy had failed, and many issues still needed to be solved [8]. In 2010, the Government of Canada passed Bill 5-31 titled An Act Respecting the Safety of Drinking Water [8]. Bill 5-31 was promptly rejected by the Chiefs of Ontario [8]. Bill 5-8, a revision of Bill 5-31, claimed that the federal government would not ‘abrogate or derogate from any existing Aboriginal or Treaty rights of the Aboriginal people of Canada under Section 35 of the *Constitution Act, 1982*… except to the extent necessary to ensure the safety of drinking water on First Nations land’ [8].^7^ Bill 5-8 was rejected. In June 2013, the federal government brought forward the Safe Drinking Water for First Nations Act [8]. The Act suggests that all water, whether on Indigenous or non-Indigenous land, should be of equal quality [8]. The Chiefs of Ontario noted that there are problems with the Act insofar that the federal government did not provide sufficient funding for water infrastructure to First Nations communities. As this Act is a senate bill, there was no funding attached [26]. Therefore, there are no supplies given to increase the capacity of drinking water – there are only ideas and possible financial stress to realise the goals of the Act. Furthermore, there are potential concerns about Treaty rights being ignored [8]. For example, in a consultation done by Koch Thornton LLP, it was suggested that even if current government acts with the Chiefs, this does not mean that other, future governments, will do the same: ‘Even in a best-case scenario where regulations are developed in full collaboration and implementation assigned to First Nation bodies, the fact that the statute itself has no structure means a future government can purport to re-assign authority and re-write the rules of engagement without debate or consultation’ [26]. This is problematic and is emblematic of the problem of non-commensurable values mentioned earlier. If Government A respects First Nations peoples’ rights to water governance, it is not the case that Government B will. As the federal government, in the case of the Safe Drinking Water Act, can override past Treaty agreements under the guise of protection it is hard to conceive of the Safe Drinking Water Act as being entirely beneficial for Indigenous communities. In reconciling this case, the federal government can look to New Zealand and its treatment of Te Awa Tupua and the Whanganui River Act, where

The innovative dreams and actions of the iwi (tribes) at the heart of these places – Whanganui Iwi and Ngāi Tuhoe – along with the [Canadian government], are positively transformative landmarks for us as a nation. These statutes, and other Treaty of Waitangi settlement statutes, endorse Māori tribal visions for knowing and caring for lands and waters and reassert a founding place for tikanga Māori (Māori law) for guiding regional natural resource governance and management [27].

Within Māori law, similar to First Nations law, there is an ethic of responsibility to ‘do things in the “right” way’ [27]. One example of acting in the ‘right’ way with water is not mixing human waste and drinking water [27]. As such, in respecting the Māori and giving rights to the water, the government in New Zealand has conceived of the human–water relationship in a way that the Government of Canada has failed to do.

### Chiefs of Ontario 2008 Water Declaration of the Anishinaabek, Mushkegowuk, and Onkwehonwe

Frustrated by the provincial and federal governments’ governance of the Great Lakes, the Chiefs of Ontario sought to change existing policy [20]. The Chiefs of Ontario attacked policy at three different levels: (1) international – Great Lakes Water Quality Agreement; (2) national – Canada–Ontario Agreement Respecting the Great Lakes Water Quality Agreement; and (3) provincial – Great Lakes Strategy and Great Lakes Protection Act [20]. In 2008, after years of deliberation amongst Indigenous Elders, the Chiefs of Ontario presented the Water Declaration of the Anishinaabek, Mushkegowuk, and Onkwehonwe [20]. The Declaration, which emphasises Indigenous ways of knowing, ‘resulted in recognition of Traditional Knowledge in the *Great Lakes Protection Act, 2015*’ [20]. The purpose of the Great Lakes Protection Act is two-fold: to restore the ecological health of the Great Lakes–St. Lawrence River Basin and to increase community participation in ecological restoration [28]. The Act also declares: ‘For greater certainty, nothing in this Act shall be construed so as to abrogate or derogate from the protection provided for the existing aboriginal and treaty rights of the aboriginal peoples of Canada as recognised and affirmed in section 35 of the *Constitution Act, 1982*’ [28]. The goal of the Act, then, is to respect the rights of the First Nations communities that live in the surrounding Great Lakes area without infringing on past treaty rights. Further, the Chiefs of Ontario created the Great Lakes Guardian Council, a group of Indigenous and Western peoples who inform Great Lakes policy in both Canada and the United States [20]. During the inaugural meeting of the Great Lakes Guardian Council in 2016, the group recognised ‘Indigenous perspectives on what is needed to protect and restore the water. The gifts, tools, and skills that each person at the Council meeting brings to the table were acknowledged. Great Lakes restoration was described as a cross-cultural effort’ [29]. This recognition is reflective of Two-Eyed Seeing, in that members of the Great Lakes Guardian Council come from a variety of nations (e.g., M’Chigeeng First Nation, Anishinabek Nation, Mississaugas of the New Credit First Nation, Chippewas of the Thames First Nation, Settlers) and industry backgrounds (e.g., Ministry of Transportation, Ministry of Environment, McMaster University, Ministry of Energy, Ontario Ministry of Municipal Affairs and Housing). How members have come to understand and be in relation to water is representative of both Indigenous and Western ways of knowing. Heightened success came in 2017 when the Government of Canada dedicated funding to create a Great Lakes Indigenous Fund – an initiative to support Indigenous community-based knowledge praxis to help ‘protect the Great Lakes’ [20]. Institutions not associated with Indigenous nations and provincial governments are unable to get funding from the Great Lakes Indigenous Fund. However, worries arise as the decisions for projects to be selected for funding come from Environment and Climate Change Canada, a Canadian government organisation [30].

### The Community of Black Tickle, Newfoundland and Labrador

The community of Black Tickle, Newfoundland and Labrador is an Indigenous land reserve created by the Government of Newfoundland and Labrador as part of their plan to centralise and resettle Indigenous communities [2]. Without robust water infrastructure such as pipes and clean drinking water, the community relies on a portable water dispensing unit (PWDU) [2].^8^ The cost of the PWDU, approximately $2/L, makes it inaccessible to many low-income community members [2]. Distrust of the PWDU arose in the community due to its proximity to a cherished brook and the lack of funds provided by the government to sustain it [2]. The poor placement of the PWDU means that residents often have to travel by vehicle to reach their water resources – minimising accessibility for people who cannot walk long distances (∼1–2 km) [2]. Many of the residents in Black Tickle have turned to making their own water wells instead of using the PWDU. However, these wells often become contaminated with animal waste or buried in large snowfalls [2]. The failure to address water policy concerns created immeasurable negative public health outcomes. In 2014, after a request to the Government of Newfoundland and Labrador to fix the water infrastructure issues, residents were told they needed to put forward 30% of the overall costs ($8601.77) and the province would put forward 70% ($20,070.69) [2]. The province’s 70/30 cost split was inconceivable for the residents as many are of lower socioeconomic status – barely able to afford the water in the PWDU [2]. Much to the chagrin of the Black Tickle residents, the citizens of Pigeon Cove, a nearby non-Indigenous community, received $100,000 in funding when it was found out that their water pipes were contaminated during a similar time period [2]. Appreciating these three case studies, an analysis can be done to provide a call-to-action to decolonise water.

## Call-to-action

Yates et al. [32] note that in Canada, ‘[Indigenous] Elders felt current government initiatives around water to be limited and short-sighted. When we consider water, one must consider all that water supports and all that supports water’ (clarification added; [32]). Instrumental to my paper has been an ethic of responsibility and recognition. In the above case studies, there were few instances where Indigenous peoples achieved respect or recognition in water governance. Instances of secured respect for Indigenous communities came after self-advocation and preservation from Elders. Mohawk scholar Audra Simpson labels these acts of agency as refusals [33]. Refusals are actions that reflect Indigenous agency to reject state paternalism of resource use or governance (for more on acts of refusals, resurgence practices or disruptions, which aim to increase the capacity of Indigenous folk to self-determine, see [34–36]). If we ought to understand Indigenous communities as having a right to autonomous action and recognition, then it is necessary to embrace their perspectives. To explain how Indigenous perspectives could enhance water policy, I will critique OECD Principles Nine and Four using the above case studies.

### Principle Nine

Residents of Black Tickle suffered after attempting to advocate for their survival – lacking funds to meet the province of Newfoundland and Labrador’s demands. According to the Unama’ki Institute of Natural Resources (UINR), ‘Netukulimk [the ethic of responsibility] is achieving adequate standards of community nutrition and economic well-being without jeopardising the integrity, diversity, or productivity of our environment’ [37]. The Government of Newfoundland and Labrador, splitting the cost of repairing water infrastructure, denied the community of Black Tickle an ethic of responsibility. The Government of Newfoundland and Labrador increased the tension that exists between Settlers and Indigenous nations by providing a non-Indigenous community $100,000. Black Tickle residents, resorting to creating wells in the ground, harmed community nutrition and economic well-being. The community of Black Tickle showcases a fundamental flaw in Principle Nine of the OECD. The Government of Newfoundland and Labrador, maintaining a division of respect between how it treats Indigenous and non-Indigenous communities, has shown a lack of ‘trust in decision making’ [1].

The case studies in the sections Key developments in Canadian policy and legislation concerning First Nations drinking water quality from 2003 to 2013 and The Community of Black Tickle, Newfoundland and Labrador show that there is a pronounced distrust such that the Chiefs of Ontario believe the Government of Canada lacks the foresight to adhere to Treaty rights. Shortage of trust is prominent in The Community of Black Tickle, Newfoundland and Labrador section such that the Province of Newfoundland and Labrador built a PDWU near a water resource that is cherished by the community of Black Tickle. Ignoring socio-cultural inferences from the community that you are working with (i.e., learning what the community views as important concerning their cultural values), is in direct violation of an ethic of responsibility and respect. Learning what the community values when making water policy would be supported by the pillars of the OECD which foster trust and engagement. This problem speaks to the issue of non-commensurability highlighted at the beginning of the paper. If policymakers cannot understand non-numerical values, then there is a question of whether they can adequately govern water or other natural resources effectively.

### Principle Four

It is not obvious that non-Indigenous government authorities can meet complex water governance challenges in Indigenous spheres. To meet the goals of Principle Four, a government must be able to work with all of its members. For example, the section Key developments in Canadian policy and legislation concerning First Nations drinking water quality from 2003 to 2013 shows the inability of the Canadian government to create a Bill that satisfies the needs of Indigenous communities. In the section Chiefs of Ontario 2008 Water Declaration of the Anishinaabek, Mushkegowuk, and Onkwehonwe, Indigenous communities had to advocate for themselves and preserve their autonomy to create adequate governance groups in the Great Lakes area. The aforementioned is often to the detriment of Indigenous communities as the groups that are formed have to take funding from non-Indigenous sources. Thus, even in areas of self-advocacy, Settlers influence Indigenous funding. Within governance spheres where Indigenous ways of knowing are prevalent, it could be the case that Western systems infiltrate the direction of a project to maintain funded support. Specific concerns that ought to be taken into consideration in the future are whether or not the Canadian government will try to steer Indigenous programmes within WaSH. Referring back to Yates et al.’s [32] sentiment: all who are supported by water in Canada are not being supported – especially in a way that is competent, transparent or with integrity. A challenge that remains in Canadian WaSH contexts is considering Indigenous voices as equal in governance and to further address the issues of non-commensurability. To open possibilities for mitigating the above issues, research methodologies such as Two-Eyed Seeing must be given equal weight in shaping policy. Federal and provincial governments need to look to past treaty agreements and reformulate WaSH principles with Indigenous perspectives, giving Indigenous communities respect and recognition.

## Conclusion

C-24 Chair Ambassador Keisha McGuire proclaimed, in February of 2021, that, ‘This year we entered the first year of the Fourth International Decade for the Eradication of Colonialism… I call on member states to renew their commitment, to strive to make this the last decade to be observed’ [38]. I have presented this paper as *a* step out of many toward decolonising water governance in Canada. I hope to have shown in this paper that it is necessary to make space for other voices in water governance. To show the above, I provided three different case studies that relayed various successes of Indigenous organisations, advocating for their voices to be heard. Finally, I provided a brief analysis that assessed the dangers of two OECD Principles of Water Governance that the Canadian government recognises as legitimate. Three lessons were apparent by highlighting the dangers in the case studies: (1) there needs to be an addition of Indigenous Two-Eyed Seeing in water governance; (2) Canada must strengthen its nation-to-nation praxis with Indigenous communities; and (3) there needs to be a creation of space in WaSH that fosters Indigenous voices so that there can be equal participation in policy conversations to mitigate existing problems and explore new possibilities.

## Data Availability

Data sharing not applicable to this article as no datasets were generated or analysed during the current study.

## Notes

1 Similar to the commitment made by Naiomi Metallic in her work (Metallic, 2022), I recognise that the titles First Nations, Métis and Inuit are colonial in nature and are umbrella terms for Indigenous peoples in Canada, rooted in the Constitution Act, 1982. Therefore, when appropriate, I will refer to Indigenous communities by their own names (e.g., Anishinaabek, Mushkegowuk and Onkwehonwe).

2 There are problems in terms of how the provinces view the personhood/autonomy of Indigenous communities and their legitimate claims to water. For an in-depth discussion about of this, see [7].

3 For a more quantitative analysis of problematic human–environment interaction, see [15]. In this article, Vörösmarty et al. look at non-human and human environmental incidences. Incidences are described as ‘exposure to a diverse array of stressors at a given location [such as pollution or the mismanagement of water]’ (Clarification added; [15]).

4 For an international example of Indigenous voices influencing Australian policy in digital spaces, see [17].

5 For more information of Indigenous/Western collaborations in WaSH, see: Ohneganos Let’s Talk Water – a research program led by Mohawk scholar Dr. Dawn Martin-Hill (McMaster University) [19].

6 An alternative model of knowledge that fosters Indigenous/Western collaboration, separate from Two-Eyed Seeing, is the co-creation model [21]. Alternatively, another article that explores integrating Indigenous knowledge systems with Western knowledge systems is [22].

7 Section 35 of the Constitution Act, 1982 supposedly recognises and affirms the rights and autonomy of Indigenous peoples Canada [24]. For more information about the purpose of Treaties between Indigenous and non-Indigenous nations, see [25]. In this article, Dorries explores how there are hidden and obfuscated racial logics in policy implementations on behalf of the Canadian government [25]. Dorries also provides in in-depth look at the goal of Treaties in Canadian contexts [25].

8 In this section I investigate the social and health impacts of the PWDU. For information about the engineering aspects of a PWDU see [31].
